# Filiation delusion: A rare presentation

**DOI:** 10.1192/j.eurpsy.2021.1274

**Published:** 2021-08-13

**Authors:** A.C. Rodrigues, C. Oliveira

**Affiliations:** 1 Unidade De Reabilitação, Centro Hospitalar Psiquiátrico de Lisboa, Lisboa, Portugal; 2 Clínica 3, Centro Hospitalar Psiquiátrico de Lisboa, Lisboa, Portugal

**Keywords:** insight, filliation delusion, schizophrénia

## Abstract

**Introduction:**

The filliation delusion was first described in 1950 by Ey as a false belief about belonging to a family group other than one’s own. Since then, 70 years have passed and litterature is still scarce on this type of presentation.

**Objectives:**

Using a case report as a starting point, the aim of this article is to review data on the various frameworks of delusional development, while discussing in what capacity social cognition impairment, theory of mind and overall lack of insight, typical in schizophrenic patients, could be related to this type of delusion.

**Methods:**

The authors present a case report of an episode of a filliation delusion in a patient with chronic schizophrenia. A search on PubMed and ClinicalKey was performed, from which the relevant publications were selected and reviewed.

**Results:**

The case referes to a 64 year old woman previously diagnosed with schizophrenia who developed, over the period of two years, a filliation delusion. The patient believed having been born in Russia and being subsequently adopted by different families. There was history of irregular attendance to consultations and non-compliance to treatment.

**Conclusions:**

There is still lack of proper investigation regarding the development of delusions in schizophrenic patients. Social cognition and insight are important predictors of functioning, and might behave as a marker of liability to psychosis. This should have strong implications in these patients’ treatment approaches. The lack of consensual measurement instruments make it difficult to draw solid conclusions, and this should be the main focus moving forward.

